# Predicting monotherapy resistance risk in patients with low-risk gestational trophoblastic neoplasia: integration of the systemic immune−inflammation index and the prognostic nutritional index

**DOI:** 10.3389/fonc.2024.1368543

**Published:** 2024-10-01

**Authors:** Tianfu Lin, Caijin Wu, Meilian Peng, Lihua Chen, Wenyu Lin, Meijin Zheng, Qibin Wu, Pengming Sun

**Affiliations:** ^1^ Laboratory of Gynecologic Oncology, Fujian Maternity and Child Health Hospital, College of Clinical Medicine for Obstetrics & Gynecology and Pediatrics, Fujian Medical University, Fuzhou, Fujian, China; ^2^ Department of Gynecology, Zhangzhou Affiliated Hospital of Fujian Medical University, Zhangzhou, Fujian, China; ^3^ Department of Obstetrics and Gynecology, Peking Union Medical College Hospital, Chinese Academy of Medical Sciences and Peking Union Medical College, National Clinical Research Center for Obstetric and Gynecologic Diseases, Beijing, China; ^4^ Department of Gynecology, Fujian Maternity and Child Health Hospital College of Clinical Medicine for Obstetrics and Gynecology and Pediatrics, Fujian Medical University, Fuzhou, Fujian, China; ^5^ Fujian Key Laboratory of Women and Children’s Critical Diseases Research, Fujian Maternity and Child Health Hospital (Fujian Women and Children’s Hospital), Fuzhou, Fujian, China; ^6^ Fujian Clinical Research Center for Gynecological Oncology, Fujian Maternity and Child Health Hospital (Fujian Obstetrics and Gynecology Hospital), Fuzhou, Fujian, China

**Keywords:** gestational trophoblastic neoplasia, systemic immune-inflammatory index, prognostic nutritional index, low-risk, chemotherapy resistance

## Abstract

**Purpose:**

Currently, there are no reliable indicators for the early identification of patients with low-risk gestational trophoblastic neoplasia (GTN) who develop resistance to monotherapy. This study aimed to evaluate the effectiveness of combining the Systemic Immune-Inflammation Index (SII) and Prognostic Nutritional Index (PNI) in detecting early resistance to monotherapy in patients with low-risk GTN.

**Methods:**

This retrospective study included 91 patients with low-risk GTN who received initial monotherapy at Fujian Maternal and Child Health Hospital between 2013 and 2021. The SII and PNI before chemotherapy were calculated from prechemotherapy peripheral blood samples, with cut-off values determined by receiver operating characteristic (ROC) curves. The SII-PNI score ranged from 0 to 2 points and was categorized as follows: a score of 2 points indicated a high SII (≥467.02) and a low PNI (≤51.35); a score of 1 point indicated either a high SII or a low PNI; and a score of 0 points indicated neither a high SII nor a low PNI.

**Results:**

Ninety-one patients with low-risk GTN underwent monotherapy, 19 of whom developed resistance, whereas the remaining 72 did not. The SII was significantly greater in chemotherapy-resistant patients than in non-resistant patients (P=0.04), whereas the PNI was markedly lower in chemotherapy-resistant patients (P=0.002). Univariate analysis revealed that cut-off values of 467.02 for the SII (P=0.04) and 51.35 for the PNI (P=0.024) were associated with chemotherapy resistance in patients with low-risk GTN. As the SII-PNI score increased, the proportion of chemotherapy-resistant patients increased (P<0.001), and the time for human chorionic gonadotropin (hCG) normalization correspondingly increased (P<0.001). Multivariate logistic regression analysis indicated that a high SII-PNI score is an independent risk factor for chemotherapy resistance in patients with low-risk GTN (P=0.001).

**Conclusion:**

A high SII and low PNI are linked to chemotherapy resistance in patients with low-risk GTN. The pretreatment SII-PNI score is a key indicator for predicting the sensitivity of patients with low-risk GTN to single-agent chemotherapy, aiding in the early identification of individuals at high risk of resistance.

## 1 Introduction

Gestational trophoblastic neoplasia (GTN) comprises a group of rare gynaecologic malignancies associated with pregnancy, including invasive mole (IM), choriocarcinoma (CC), placental site trophoblastic tumour (PSTT), and epithelioid trophoblastic tumour (ETT) (1, 2). Owing to the insensitivity of PSTTs and ETTs to chemotherapy, surgical intervention is the primary treatment modality (2), which is not discussed in this paper. Currently, GTN is the only pregnancy-related gynaecologic malignancy that can be clinically cured through chemotherapy. Chemotherapy options include single-agent and combination therapies. On the basis of the risk of resistance to single-agent chemotherapy, patients with GTN were stratified into low-risk (≤6 points or ≤ Stage III) and high-risk (≥7 points or Stage IV) groups according to the International Federation of Gynecology and Obstetrics (FIGO) prognostic scoring (2000) and anatomical staging system. The low-risk group received single-agent chemotherapy, whereas the high-risk group received combination chemotherapy (3, 4). Although the majority of patients achieve clinical remission with chemotherapy regimens guided by the scoring system, approximately 25–30% of low-risk patients develop resistance to initial single-agent chemotherapy, particularly those with FIGO scores of 5–6, for whom the resistance rate can reach 70–80% (5–7). Although switching to another chemotherapy drug or adopting combination therapy after resistance occurs can provide relief, the extended treatment duration and drug toxicity associated with combination therapy may adversely affect patients’ quality of life (8, 9). However, there is currently a lack of reliable indicators or biomarkers to predict the response of patients with low-risk GTN to single-agent chemotherapy.

The inflammatory microenvironment within a tumour can influence the response of tumour cells to anticancer drugs, and this local microenvironment can be reflected by the systemic inflammatory response (10, 11). Previously, certain blood-based inflammatory biomarkers, such as neutrophil and lymphocyte counts, have been associated with cancer progression and prognosis. Among these biomarkers, the neutrophil−lymphocyte ratio (NLR), lymphocyte−monocyte ratio (LMR), and others were confirmed to be related to chemotherapy resistance in a recent study on GTN (12, 13). The Systemic Immune-Inflammation Index (SII) is a novel inflammation index calculated from peripheral blood neutrophil, platelet, and lymphocyte counts. Previous studies have indicated that the SII can predict the prognosis of various malignant tumours, such as gastric cancer and non-small cell lung cancer, after surgery. Additionally, the SII has been recognized for its ability to predict chemotherapy sensitivity after gastric cancer surgery (14, 15). The nutritional status of patients during chemotherapy also influences their tolerance to adverse drug reactions (16). Therefore, nutritional status during treatment is a crucial factor affecting the chemotherapy response. The Prognostic Nutritional Index (PNI) is a nutritional assessment method calculated from peripheral blood albumin levels and lymphocyte counts. Owing to its simplicity and feasibility, the PNI is widely used to predict the prognosis of various malignant tumours (16, 17). Additionally, Ding et al. reported that the pretreatment SII-PNI score is a good indicator for predicting the sensitivity of patients to immunotherapy combined with chemotherapy and prognosis of locally advanced gastric cancer (15). However, to date, the SII-PNI score has not been applied to predict the occurrence of resistance to single-agent chemotherapy in patients with low-risk GTN.

Therefore, this study aimed to assess the value of the prechemotherapy SII-PNI score in predicting chemotherapy resistance in patients with low-risk GTN receiving single-agent chemotherapy. This study aimed to facilitate the early identification of chemotherapy resistance in patients with low-risk GTN, thereby reducing toxic side effects, shortening treatment duration, and improving patients’ quality of life.

## 2 Materials and methods

### 2.1 Population

This retrospective study included 137 patients diagnosed with GTN at Fujian Provincial Maternity and Child Health Hospital between January 2013 and December 2021. Among these patients, 91 patients classified with low-risk GTN and treated with single-agent chemotherapy were included in the study. The inclusion criteria were as follows: (1) laboratory-confirmed GTN or histopathologically diagnosed CC or IM; (2) a FIGO prognostic score of 0-6 points and anatomical stage I–III; (3) absence of severe rheumatic immune diseases, blood disorders, or other chronic debilitating conditions; (4) no prior treatment other than surgical intervention before chemotherapy; and (5) completion of the full course of chemotherapy with regular follow-up, including at least three consecutive negative human chorionic gonadotropin (hCG) results sustained for at least three months. The exclusion criteria were as follows: (1) histopathological confirmation of PSTT or ETT; (2) a FIGO score ≥7 points or anatomical stage IV; (3) prior immunotherapy or targeted therapy before chemotherapy; (4) change in chemotherapy drugs due to adverse effects; and (5) missing data before chemotherapy, incomplete chemotherapy, or loss to follow-up after completing chemotherapy. The study was approved by the Ethics Committee of the College of Clinical Medicine for Obstetrics & Gynecology and Pediatrics, Fujian Medical University (FMCH2021KRD020). Informed consent was obtained from all patients.

### 2.2 Definitions

Before the first cycle of chemotherapy, peripheral venous blood samples were collected from fasted patients for analysis, including complete blood cell analysis and routine biochemical examinations. Additionally, ultrasound or imaging evaluations were performed on the pelvic, thoracoabdominal, and cranial regions. The peripheral neutrophil count, lymphocyte count, platelet count, and albumin level were measured and analysed via an automated haematology analyser. The PNI and SII were defined as follows: PNI = Albumin (g/L) + 5 × Total Lymphocyte Count (10^9/L); SII = Platelet Count × Neutrophil Count/Lymphocyte Count (18).

### 2.3 Chemotherapy regimen and assessments

Patients underwent a single-agent chemotherapy regimen with either methotrexate (MTX) or actinomycin-D (Act-D). In the MTX regimen, patients received 1 mg/kg MTX intramuscularly on days 1, 3, 5, and 7, along with 0.1 mg/kg folinic acid intramuscularly on days 2, 4, 6, and 8, with cycles repeated every two weeks. Alternatively, patients received 1.25 mg/m² actinomycin-D intravenously (up to a maximum of 2 mg) every two weeks. During chemotherapy, blood hCG levels were monitored weekly, and before each cycle, the pelvic, thoracoabdominal, and cranial regions were evaluated via ultrasound or imaging studies. If, after two consecutive chemotherapy cycles, hCG levels did not show a logarithmic decline or plateau (decrease <10%), or if imaging studies revealed that the tumour lesions did not shrink, the lesions increased in size, or new lesions appeared, the patient was considered resistant to single-agent chemotherapy. In cases of resistance, if the hCG level plateaued and was <300 U/L, a switch to alternative single-agent chemotherapy was considered. If the hCG level plateaued at >300 U/L, the hCG level increased, new lesions appeared, or there was a poor response to both single-agent chemotherapies, combination chemotherapy was initiated. Patients who did not develop resistance continued consolidation chemotherapy for 2–3 additional cycles after their hCG levels normalized (19).

### 2.4 Statistical analyses

All the statistical analyses were conducted via SPSS software (version 27.0), GraphPad Prism (version 8.0), and R language (version 4.2.2). Receiver operating characteristic (ROC) curve analysis was performed to identify the optimal cut-off values for the SII and PNI. The value closest to the maximum sum of the sensitivity and specificity (Youden index) was considered the optimal cut-off. The optimal cut-off values were identified as 467.02 for the SII and 51.3 for the PNI. The correlation between the PNI and the SII was assessed via Spearman correlation analysis. The associations of the SII, PNI, and other clinical indicators with chemotherapy resistance were analysed via chi-square tests and Fisher’s exact tests. Univariate and multivariate analyses were performed via logistic regression models, with hazard ratios (HRs) and 95% confidence intervals (CIs) calculated to assess relative risk. P values ≤0.05 were considered statistically significant.

## 3 Results

### 3.1 Patient demographic information and clinical features

**Figure 1:**
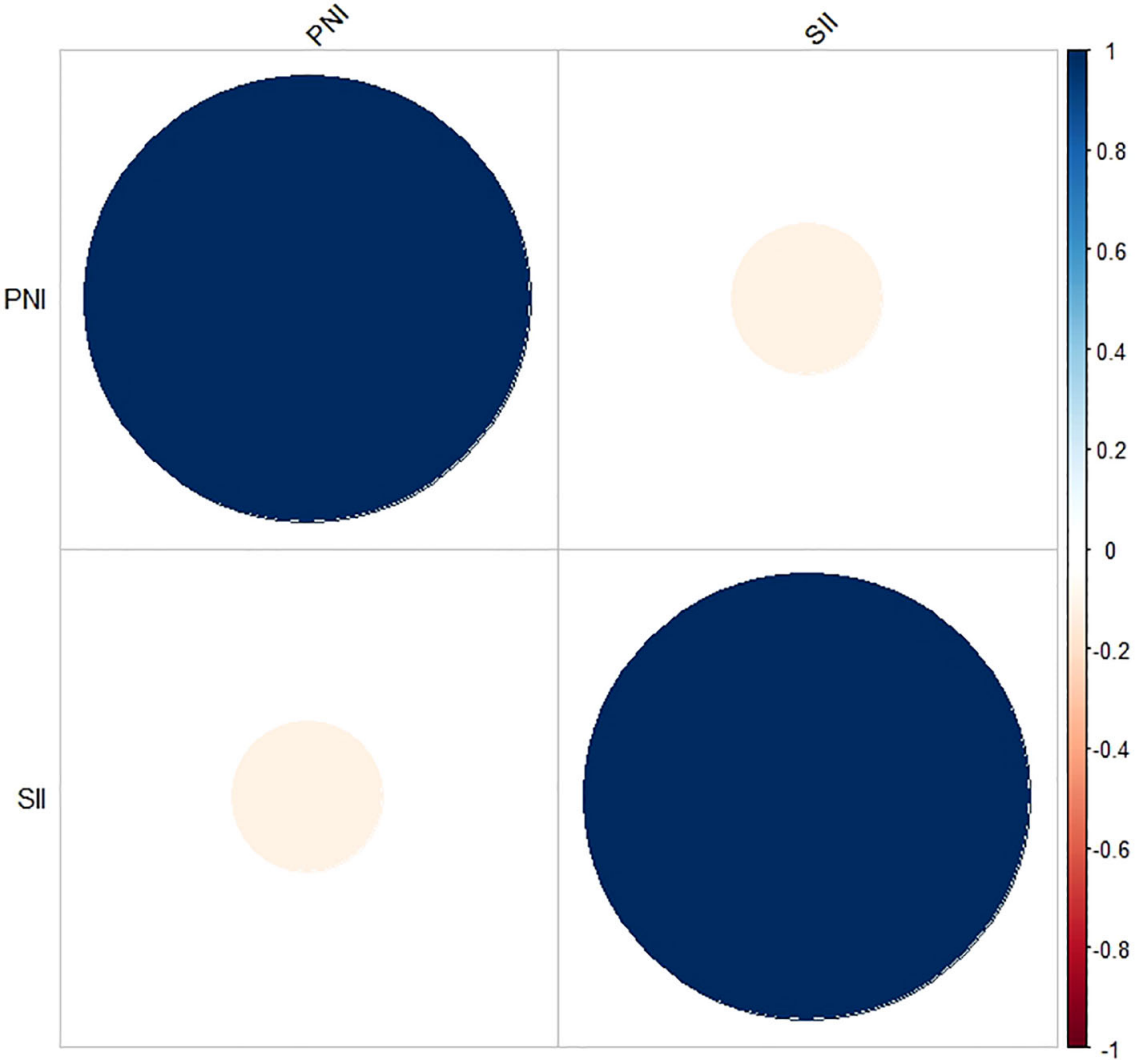
Correlation analysis between SII and PNI.

This study retrospectively included 91 patients diagnosed with low-risk GTN. The demographic characteristics and clinical features of these patients are summarized in **Table 1**. The median age of the patients was 29 years, with a range of 16 to 54 years. A total of 40 patients (44%) were in anatomical stages I and II, whereas 51 patients (56%) were in stage III. FIGO scores ranged from 0-4 points in 66 patients (73%) and from 5-6 points in 25 patients (27%). The SII ranged from 150.27 to 1961.58, and the PNI ranged from 44.35 to 58.70. The median values of the pretreatment SII and PNI were 468.41 and 50.50, respectively. Additionally, there was no significant correlation between the SII and PNI (r = -0.115, P = 0.276; **Figure 1**).

**Table 1:** Individual and clinical characteristics of low-risk GTN patients in fujian provincial maternal and child health hospital (* and **are determined based on the FIGO 2000 anatomical staging and prognostic scoring assessment).

Clinical and therapeutic characteristics	Median (interquartile range)/%
Age(years)	29(25, 38)
BMI	20.63(19.19,22.66)
Gravidity	2(1,4)
Parity	1(0, 1)
origin of gestational trophoblastic neoplasia	
Molar	73(80%)
Abortion	16(18%)
Full term delivery	2(2%)
Time between the end of last pregnancy and beginning of chemotherapy, months	
<4	72(79%)
4-6	5(6%)
7-12	3(3%)
>12	11(12%)
Stage*	
I	38(42%)
II	2(2%)
III	51(56%)

### 3.2 Optimal cut-off values for the SII and PNI before chemotherapy

**Figure 2:**
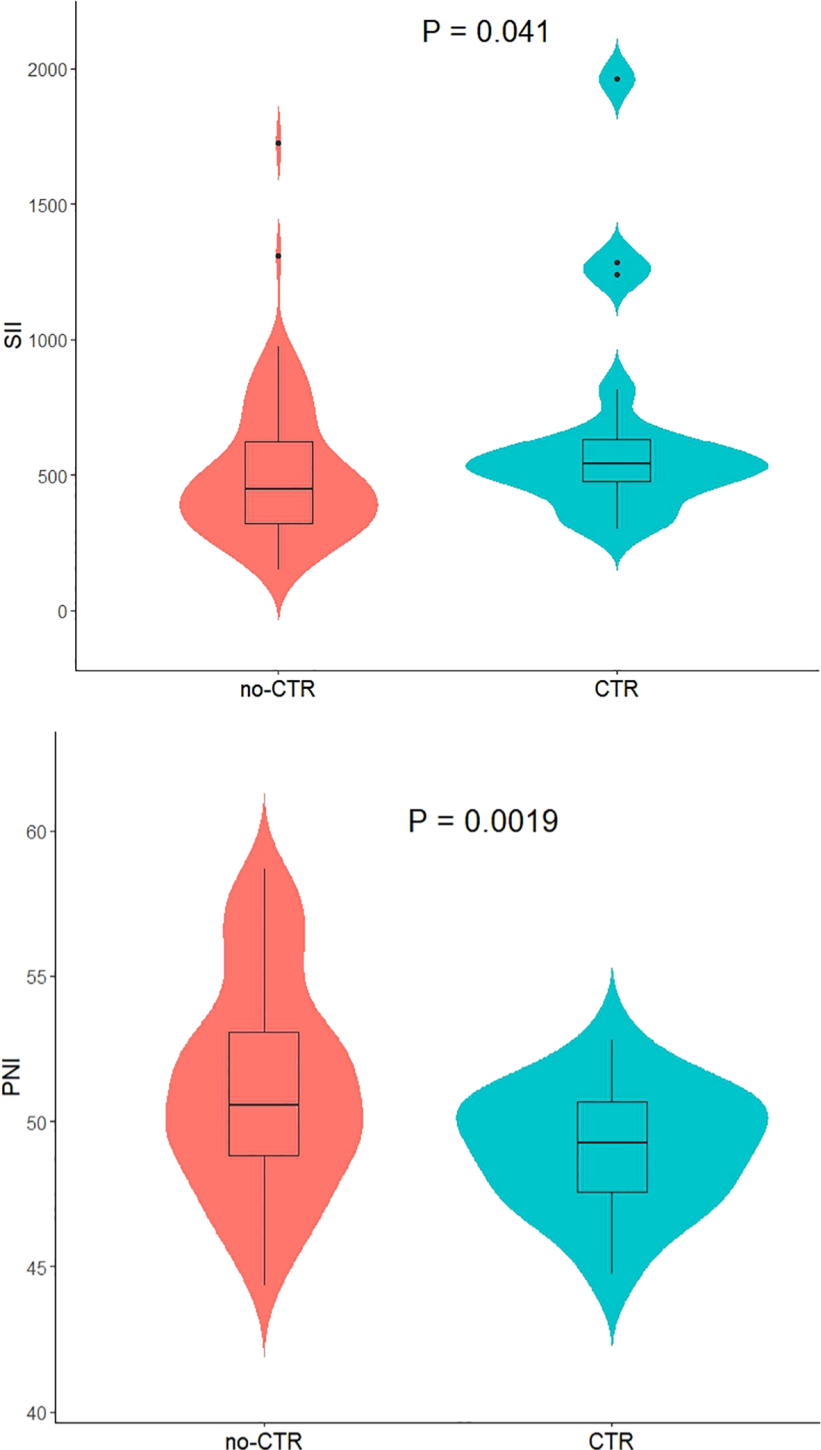
The relationship between SII /PNI and monotherapy chemotherapeutic responsiveness in low-risk GTN patients.

**Figure 3:**
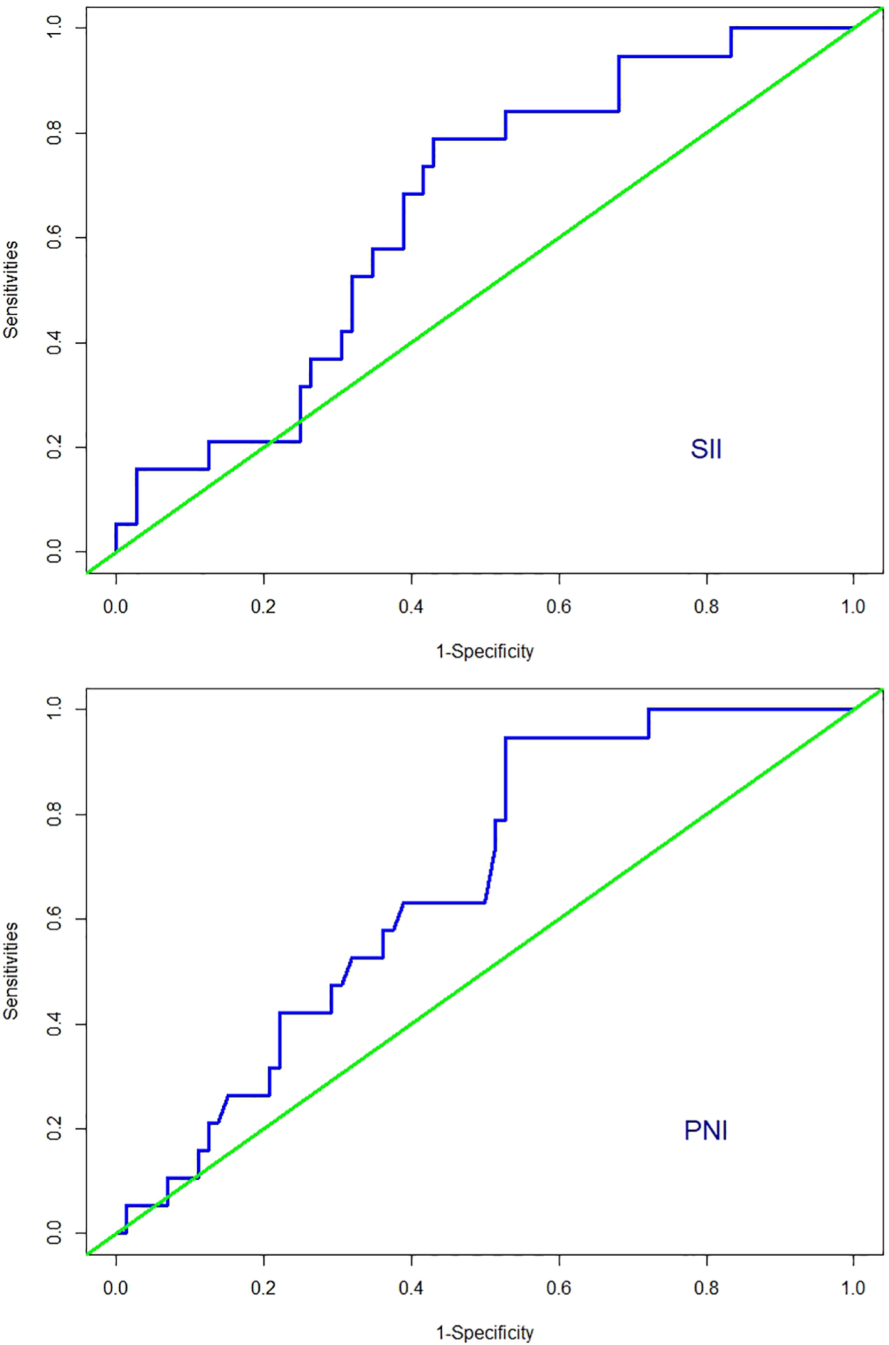
ROC curves for discriminating patients with CTR and those with no-CTR according to values of the SII and PNI.

Among the 91 patients with low-risk GTN in the present study, the median SII (interquartile range) for the 72 non-resistant patients was 446.57 (317.60, 658.37), and the mean ± standard deviation of the PNI was 51.17 ± 3.58. For the 19 resistant patients, the median SII was 541.29 (468.41, 648.39), and the mean ± standard deviation of the PNI was 49.10 ± 2.06. The SII in resistant patients was significantly greater than that in non-resistant patients (P=0.04; **Figure 2**), whereas the PNI in resistant patients was significantly lower than that in non-resistant patients (P=0.0019; **Figure 2**). Further analysis via ROC curve calculations was used to determine the cut-off values for the SII and PNI between the resistant and non-resistant groups. The cut-off value for the SII was 467.02 [AUC = 0.654, 95% CI 0.529–0.778, P = 0.04], with a sensitivity of 0.789 and specificity of 0.569 **Figure 3**. The cut-off value for the PNI was 51.35 [AUC = 0.668, 95% CI 0.551–0.786; P = 0.024], with a sensitivity of 0.472 and specificity of 0.947 (**Figure 3**). Scores were assigned on the basis of the cut-off values: an SII ≥ 467.02 was given 1 point (high SII), otherwise, 0 points were given; a PNI ≤ 51.35 was given 1 point (low PNI), otherwise 0 points were given. Patients were categorized into three groups according to the assigned score (SII-PNI score): 2 points (n=30, high SII + low PNI), 1 point (n=39, either high SII or low PNI), and 0 points (n=22, neither high SII nor low PNI).

### 3.3 Relationship between the prechemotherapy SII-PNI score and chemotherapy resistance

Initially, all patients received monotherapy until clinical evidence of chemotherapy resistance emerged. Among these patients, nineteen (21%) developed resistance, whereas seventy-two (79%) did not. Among the nineteen patients who developed resistance, seven were switched to the EMA-CO regimen, nine were switched to the 5-Fu+Act-D regimen, and three were switched to alternative single-agent chemotherapy. After the chemotherapy regimen was changed, all fifteen patients achieved clinical remission. However, four patients initially treated with the 5-Fu+Act-D regimen developed resistance again. These four patients were subsequently switched to the EMA-CO regimen and ultimately achieved clinical remission. The SII-PNI score consistently remained at 2 points for patients with both drug resistance and a FIGO score of 5-6 points. The SII-PNI score for non-resistant patients was significantly lower than that for resistant patients (P < 0.001; **Table 2**).

**Table 2:** Comparison of monotherapy response with personal and clinical characteristics in low-risk GTN patients.

Factor	Non-CTR (n=72)	CTR (n=19)	P value
Age(years)			0.631
<40	59	14	
≥40	13	5	
BMI,mediam	20.62 (19.28,22.09)	20.96 (18.73,23.69)	0.747
Gravidity,mediam	2(1,4)	3(2,4)	0.135
Parity	1(0, 1)	1(0,2)	0.085
origin of gestational trophoblastic neoplasia			1.000
Molar	57	16	
Abortion	13	3	
Full term delivery	2	0	
Interval Since Last Pregnancy, months			0.072
<4	60	12	
4-6	4	1	
7-12	1	2	
>12	7	4	
Pulmonary metastasis			0.392
Absent	30	10	
Present	42	9	
FIGO risk score			0.481
0-4	51	15	
5-6	21	4	
Pre-chemotherapy hCG, mIU/ml			0.665
<10^3	24	6	
10^3-10^4	24	8	
10^4-10^5	22	4	
>10^5	2	1	
Pre-chemotherapy SII-PNI			<0.001
0	20	1	
1	35	3	
2	17	15	

### 3.4 High-risk factors for monotherapy chemoresistance in patients with low-risk gestational trophoblastic neoplasia

On the basis of the univariate analysis and considering clinical indicators commonly believed to impact chemotherapy efficacy, five indicators—age, parity, gravidity, time since the last pregnancy, and prechemotherapy SII-PNI score—were included in the univariate logistic regression analysis. The results indicated that parity (P = 0.005, HR = 5.091) and a high the prechemotherapy SII-PNI score (P < 0.001) were significant risk factors for single-agent chemotherapy resistance in patients with low-risk GTN (**Table 3**). Further multivariate logistic regression analysis revealed that the prechemotherapy SII-PNI score (P = 0.001) independently contributed to an increased risk of single-agent chemotherapy resistance in patients with low-risk GTN (**Table 3**).

**Table 3:** Single and multifactor logistic regression analysis of chemoresistance risk factors in low-risk GTN.

Independent factor	Univariate analysis			Multivariate analysis		
	Hazard ratio	95% CI	P value	Hazard ratio	95% CI	P value
Age (years)			0.424			
<40	1.000	Reference				
≥40	1.621	0.496-5.299				
Gravidity			0.156			
≤1	1.000	Reference				
≥2	2.386	0.718-7.926				
Parity			0.005			0.057
≤1	1.000	Reference		1.000	Reference	
≥2	5.091	1.616-16.041		3.560	0.964-13.150	
Interval Since Last Pregnancy(Months)			0.171			
<4	1.000	Reference				
4-6	1.250	0.128-12.188	0.848			
7-12	10.000	0.838-119.315	0.069			
>12	2.857	0722-11.311	0.135			
Pre-chemotherapy SII-PNI			<0.001			0.001
0	1.000	Reference		1.000	Reference	
1	1.714	0.167-17.600	0.650	1.618	0.154–16.978	0.688
2	17.647	2.108-147.756	0.008	14.560	1.695-125.045	0.015

### 3.5 Relationship between the pre-chemotherapy SII-PNI Score and the time to hCG normalization after chemotherapy

**Figure 4:**
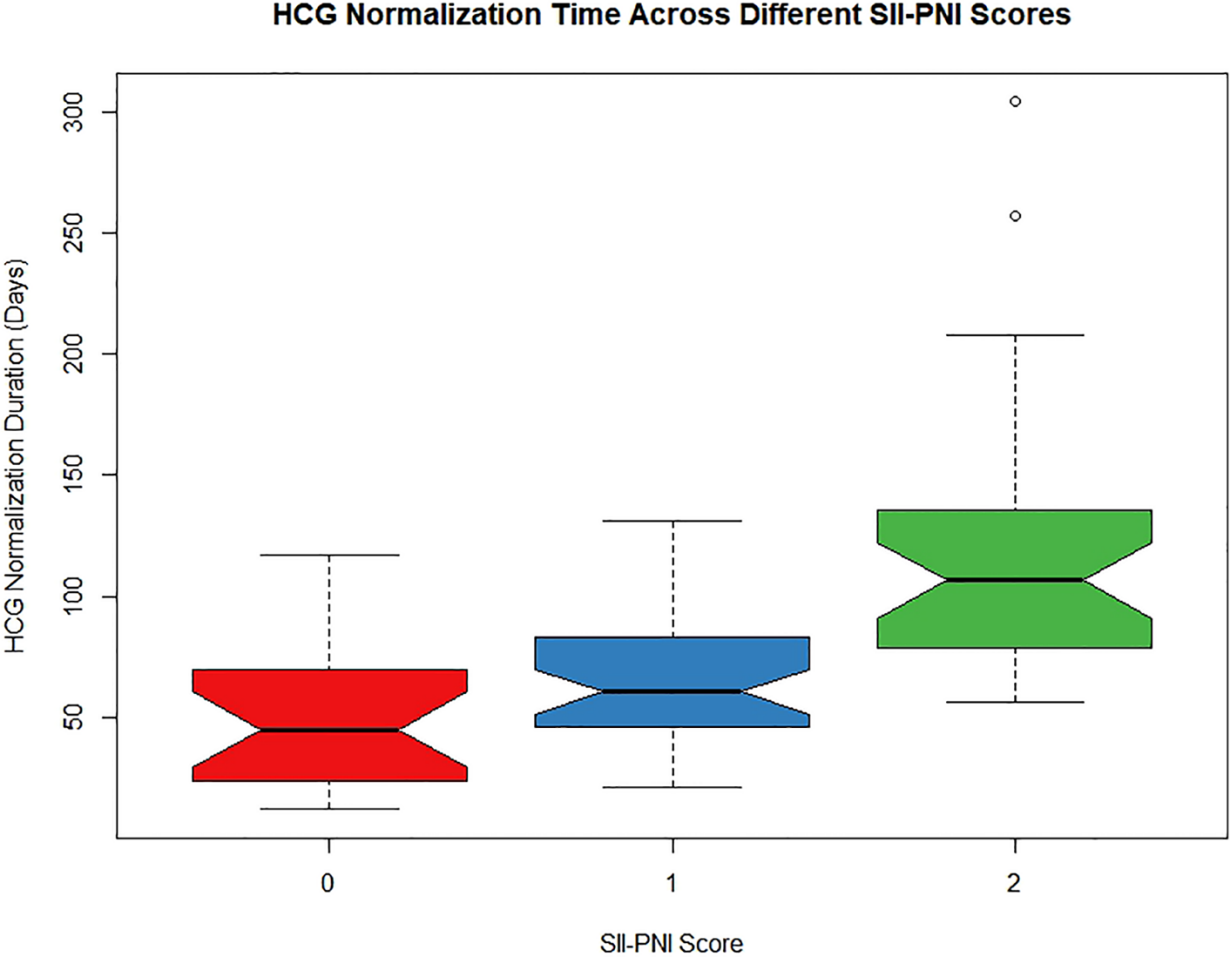
The relationship between SII-PNI score and time to hCG normalization after single-agent chemotherapy in low-risk GTN patients.

The median time for hCG normalization across all patients was 70 days. However, when the patients were stratified by the SII-PNI score (0 points, 1 point, and 2 points), the median times for hCG normalization were 45 days, 60.5 days, and 106.5 days, respectively. The results indicate that as the SII-PNI score increased, regardless of patient resistance, the time to hCG normalization significantly increased (P < 0.001; **Figure 4**). Particularly noteworthy were the significant differences observed between the groups with 0- and 2-point scores (P < 0.001) and between the groups with 1- and 2-point scores (P = 0.001).

## 4 Discussion

GTN constitutes a rare group of gynaecologic malignancies. The primary treatment modalities currently include chemotherapy, immunotherapy, targeted therapy, and surgical resection. Immunotherapy and targeted therapy are still in the early stages of clinical exploration, but surgery is generally reserved for patients with persistent lesions. Therefore, chemotherapy remains the predominant approach at present (2). In clinical practice, most patients achieve clinical remission through chemotherapy, with the cure rate approaching nearly 100% for the low-risk GTN group, as defined by FIGO scores (20). However, before achieving clinical remission, some patients with low-risk GTN may develop resistance to initial single-agent chemotherapy. Although this issue can be managed by switching to another chemotherapy drug or adopting combination chemotherapy (9), the resulting prolongation of chemotherapy courses and the toxic side effects of multiple drugs can significantly impact patients’ quality of life. Therefore, it is imperative to explore predictive factors or biomarkers that can identify patients with sensitivity to chemotherapy drugs early. This exploration is crucial for selecting appropriate treatment regimens before therapy begins and for guiding regular follow-up monitoring after treatment.

Previous studies have attempted to predict the response to first-line chemotherapy and the prognosis of GTN by combining serum peptides with FIGO prognostic scores (21). While this approach partially improved the predictive ability of the responsiveness of patients with low-risk GTN to first-line single-agent chemotherapy, it also increased patients’ medical expenses. Research has shown a relationship between the inflammatory response and the occurrence and progression of malignant tumours. The SII, which reflects the overall inflammatory status, has been shown to predict the response to chemotherapy and prognosis of various malignancies (14, 22). A patient’s nutritional status also influences their tolerance and response to chemotherapy and is a crucial factor affecting the treatment and prognosis of malignant tumours. The prognostic value of the PNI has been validated in patients with gastric cancer and other malignancies (15, 16, 23). Researchers have previously used ratios of inflammatory indicators, such as neutrophils, monocytes, and lymphocytes, to study chemotherapy resistance in patients with GTN (13). A recent study used the SII-PNI score to predict the response of patients to immunotherapy combined with preoperative neoadjuvant chemotherapy and the prognosis of locally advanced gastric cancer (15). To date, this is the first study to combine the SII and PNI to establish the SII-PNI scoring system, which can predict resistance to first-line single-agent chemotherapy and the duration of hCG normalization in patients with low-risk GTN.

The responsiveness of patients with low-risk GTN to chemotherapy is a critical factor influencing GTN prognosis. Currently, predicting the chemotherapy responsiveness of patients with low-risk GTN via clinically relevant indicators before treatment is challenging. The SII used in this study has been employed to predict whether patients with breast cancer can achieve a pathological complete response and the prognosis of breast cancer after neoadjuvant chemotherapy (24, 25). The PNI is also widely used in clinical practice to assess the efficacy of neoadjuvant chemotherapy and the prognosis of oesophageal cancer, lung cancer, and other malignancies (16, 17). However, the use of the SII and PNI in combination to predict chemotherapy responsiveness and the duration of hCG normalization after chemotherapy in patients with low-risk GTN has not yet been reported. This study analysed the relationships between prechemotherapy SII, PNI, and SII-PNI scores and the risk of drug resistance to single-agent chemotherapy in patients with low-risk GTN. The results revealed that the AUC values for the optimal cut-off points of the SII and PNI ranged from 0.65 to 0.70, indicating a strong predictive ability that closely reflects clinical reality. These findings suggest that the SII and PNI are relatively stable in predicting drug resistance in patients with low-risk GTN. Additionally, the study revealed a close relationship between the SII-PNI score and resistance to single-agent chemotherapy, with higher scores associated with a greater risk of drug resistance. This study further identified a high prechemotherapy SII-PNI score as an independent risk factor for single-agent chemotherapy resistance, suggesting that it could serve as a potential biomarker for predicting drug resistance in patients with low-risk GTN. This indicator is both cost-effective and simple, aiding in the formulation of accurate and effective treatment plans before chemotherapy. Notably, in the initial World Health Organization (WHO) prognostic scoring classification for GTN, patients with FIGO scores of 5-6 points were originally classified into the intermediate-risk group but were later classified into the low-risk group. Studies have shown that patients in this subgroup have a significantly greater probability of resistance than those with FIGO scores of 0-4 points (26). In this study, when FIGO scores of 5-6 points were added to the SII−PNI scores of patients in the low-risk group, 10 patients had total scores ranging from 7 to 8 points. Among these 10 patients, four were resistant to single-agent chemotherapy, accounting for 40% of the patients. However, this finding suggests that combining the two scores can significantly increase the predictive accuracy of resistance to single-agent chemotherapy in patients with FIGO scores of 5–6 points. This approach could facilitate early initiation of combination chemotherapy for this subgroup, avoiding prolonged chemotherapy courses and exacerbated side effects due to resistance. Additionally, some studies suggest that despite a higher incidence of resistance to single-agent chemotherapy in patients with FIGO scores of 5-6 points, there is no significant difference in the total number of treatment cycles between those who experience resistance and those who do not. Furthermore, starting with combination chemotherapy from the outset may lead to increased side effects. This suggests that for patients with FIGO scores of 5-6 points, regardless of resistance to single-agent chemotherapy, initial treatment with single-agent chemotherapy may offer greater benefits (7). However, further research is needed to confirm this observation.

This study also evaluated the relationship between prechemotherapy SII-PNI scores and the time to hCG normalization after chemotherapy. The results revealed that patients with SII-PNI scores of 0, 1, and 2 points had median hCG normalization times of 45 days, 60.5 days, and 106.5 days, respectively. This finding indicates that as the SII-PNI score increases, there is a significant delay in hCG normalization, regardless of the development of drug resistance. Since hCG levels are a reliable indicator of gestational trophoblastic disease activity, increased hCG levels reflect disease persistence, whereas decreased hCG levels indicate regression (27). Therefore, these results suggest that patients with higher SII-PNI scores may have lower tumour cell susceptibility to single-agent chemotherapy than those with lower scores. This is especially evident in patients with an SII-PNI score of 2 points, suggesting that this subgroup might benefit from early combination chemotherapy to prevent drug resistance and reduce the overall duration of treatment.

This study also assessed the relationship between the SII-PNI score before chemotherapy and the time to hCG normalization after chemotherapy. The results revealed that patients in the groups with SII-PNI scores of 0, 1, and 2 points had median times to hCG normalization of 45 days, 60.5 days, and 106.5 days, respectively. This finding indicates that as the SII-PNI score increases, regardless of the development of drug resistance, there is a significant increase in the time to hCG normalization among patients. Since hCG levels are a reliable indicator of the activity of gestational trophoblastic disease, an increase in hCG levels reflects the persistence and activity of the disease, whereas a decrease in hCG levels indicates disease regression (27). Therefore, these results suggest that patients with higher SII-PNI scores may have a lower susceptibility of tumour cells to single-agent chemotherapy drugs than those with lower SII-PNI scores. This is particularly evident in patients with an SII-PNI score of 2 points, suggesting that this subgroup of patients may benefit from early initiation of combination chemotherapy to avoid drug resistance and shorten the overall treatment duration.

However, this study has certain limitations. As a retrospective study, dividing patients into two subgroups resulted in relatively small sample sizes for each group. Therefore, patients receiving either of the two first-line chemotherapy drugs were combined for analysis. Additionally, because the AUC values for the optimal cut-off points of the SII and PNI are only within the moderate range, there are limitations in fully reflecting clinical reality. Considering the significant potential clinical benefits suggested by the study results, there is an urgent need for larger-scale retrospective studies. These studies should stratify populations on the basis of the type of first-line chemotherapy drug used to further validate these findings and increase their clinical applicability.

## 5 Conclusion

In summary, our research indicates that the SII-PNI score before chemotherapy can predict the risk of single-agent chemotherapy resistance in low-risk GTN patients, as well as the time required for hCG levels to return to normal.

## Data Availability

The original contributions presented in the study are included in the article/supplementary material. Further inquiries can be directed to the corresponding authors.

## Glossary

GTN: gestational trophoblastic neoplasia
SII: systemic immune inflammation index
PNI: prognostic nutritional index
hCG: human chorionic gonadotropin
CTR: chemotherapy resistance
no-CTR: no- chemotherapy-resistance
IM: invasive mole
CC: choriocarcinoma
PSTT: placental site trophoblastic tumour
ETT: epithelioid trophoblastic tumour
NLR: neutrophil-lymphocyte ratio
LMR: lymphocyte-monocyte ratio
MTX: methotrexate
Act-D: actinomycin-D
